# Human Elimination of Phthalate Compounds: Blood, Urine, and Sweat (BUS) Study

**DOI:** 10.1100/2012/615068

**Published:** 2012-10-31

**Authors:** Stephen J. Genuis, Sanjay Beesoon, Rebecca A. Lobo, Detlef Birkholz

**Affiliations:** ^1^Faculty of Medicine, University of Alberta, 2935-66 Street, Edmonton, AB, Canada T6K 4C1; ^2^Department of Laboratory Medicine, University of Alberta, Edmonton, AB, Canada T6G 2B7; ^3^Department of Family Medicine, University of Alberta, Edmonton, AB, Canada T6G 2C8; ^4^Environmental Division, A.L.S. Laboratory Group, Edmonton, AB, Canada T6E 5C1

## Abstract

*Background*. Individual members of the phthalate family of chemical compounds are components of innumerable everyday consumer products, resulting in a high exposure scenario for some individuals and population groups. Multiple epidemiological studies have demonstrated statistically significant exposure-disease relationships involving phthalates and toxicological studies have shown estrogenic effects in vitro. Data is lacking in the medical literature, however, on effective means to facilitate phthalate excretion. *Methods*. Blood, urine, and sweat were collected from 20 individuals (10 healthy participants and 10 participants with assorted health problems) and analyzed for parent phthalate compounds as well as phthalate metabolites using high performance liquid chromatography-tandem mass spectrometry. *Results*. Some parent phthalates as well as their metabolites were excreted into sweat. All patients had MEHP (mono(2-ethylhexyl) phthalate) in their blood, sweat, and urine samples, suggesting widespread phthalate exposure. In several individuals, DEHP (di (2-ethylhexl) phthalate) was found in sweat but not in serum, suggesting the possibility of phthalate retention and bioaccumulation. On average, MEHP concentration in sweat was more than twice as high as urine levels. *Conclusions*. Induced perspiration may be useful to facilitate elimination of some potentially toxic phthalate compounds including DEHP and MEHP. Sweat analysis may be helpful in establishing the existence of accrued DEHP in the human body.

## 1. Introduction

As a family of man-made chemical compounds, phthalates are a standard component of modern day plastics and are specifically used to create plastic products that are soft and malleable. First developed in the 1920s, some phthalates have also been found to maintain color and scent in various mediums and are thus used in a wide variety of consumer goods including fragrances, paints, and nail polish. As a result, production of phthalate compounds has exploded over the last half century and they have increasingly been incorporated into assorted household and medical materials [1]. Often referred to as plasticizers, phthalates can be found in medical devices such as intravenous tubing and blood collection bags. Moreover, they are extensively used in plastic wrapping for food and beverage packaging, and are a ubiquitous component of soft plastic toys as well as various other products including vinyl floor tiles, shower curtains, synthetic leather, cosmetics, shopping bags, and pharmaceuticals [2–5].

With the widespread use of phthalates in numerous everyday products, these compounds have become one of the most common synthetic chemical exposures, resulting in concern about the potential impact of phthalates on human health. Phthalates have recently been detected throughout large population samples in both North America and Europe, and more recently have been found in fetal samples [6–10]. Recent evidence demonstrates a link between phthalate exposure and adverse health effects in both animal and human models, raising the question of whether usage of these compounds requires regulation—a concern that recently prompted the signing of a European ban in 2005 on certain phthalates in all childcare articles and toys [11]. An overview of the literature regarding the potential effects of phthalates on human health is presented, followed by data from 20 subjects whose blood, urine, and sweat were tested for phthalate compounds.

## 2. Background

Phthalates are synthesized as an ester of benzenedicarboxylic acid (also known as phthalic acid) and are valued for their ability to promote both flexibility and stability in plastics [2]. Diisononyl phthalate (DINP), diisodecyl phthalate (DIDP), and di-2-ethyl-hexyl phthalate (DEHP) are the most common types of compounds used within the phthalate family, with DEHP representing the highest proportion of produced phthalates as a component in the mass produced plastic, polyvinyl chloride (PVC) [12, 13]. Because phthalates are not covalently bound as plasticizers, they are able to migrate from phthalate-containing items into air, dust, water, soil, and sediment, leading to widespread human exposure through ingestion, inhalation, and dermal contact [5, 14]. 

Once they enter the body, phthalates undergo a series of phase I hydrolysis and phase II conjugation reactions and are subsequently excreted in feces and urine [15]. Existing literature suggests that phthalate clearance from the body is rapid and primarily via urinary excretion with only a slight cumulative potential. Thus, the major mechanism of detection is through screening urine for monoesters [16–18]. This was originally thought to be an accurate measure of all phthalate exposure; however, recent work regarding phthalate metabolism suggests it is likely to underestimate exposure to phthalates with long alkyl chains, such as DEHP and DINP, which undergo further metabolism prior to excretion [8]. Both primary and secondary phthalate metabolites are biologically active [19–22]. Conclusive evidence on levels of phthalate bioaccumulation within specific organs and tissues of the body has not been available thus far. 

### 2.1. Human Exposure

Throughout the latter parts of the 20th century and the current 21st century, multiple urine samples analyzed from populations worldwide have consistently demonstrated phthalate exposure in up to 98% of participants, including pregnant women [6, 8–10, 23–25]. As phthalates are thought to have a relatively short half-life of less than 5 hours, this widespread detection is likely to indicate chronic exposure [15], rather than accrual within the body. 

Sources and pathways of exposure may vary widely. In neonates, infants, and toddlers, exposure may come through vertical transmission or external sources. The most likely neonatal exposure pathway is vertical transmission through the placenta or breast-feeding. In utero, phthalates circulate through the placenta and into fetal blood, where they are found to have an extended half-life as compared to maternal serum (up to 6.2 and 64 hours in fetal serum and amniotic fluid, resp.) [15, 26]. Breast milk is also found to contain detectable levels of phthalates, particularly the most hydrophobic compounds, which include DEHP and DINP [27–30]. Infant formula, baby food, and children's toys are additional sources of exposure, a realization that has prompted Europe to enact legislation limiting use of these compounds in order to prevent adverse effects in development [8, 11, 31–34]. 

Other common sources of exposure in the general population include ingestion of contaminated food and dust. Phthalates are able to easily leech from plastics into proximal food and fluids and are found at highest concentration in foods with high fat concentrations, such as dairy, poultry, and oils [8, 14, 35]. Absorption of phthalates can also occur via dermal contact [5]. This is of concern with products such as deodorant, perfumes, aftershave, hair styling products, shampoo, skin and nail care products, as well as cosmetic products—which have been found to contain varying amounts of phthalates, ranging from 1–15,000 mg/kg [8]. Additionally, neonates or children who spent time in an intensive care unit and patients who are critically ill are exposed to high levels of phthalates through medical equipment including intravenous bags and tubing [36–38]. 

### 2.2. Potential Human Health Implications

A population analysis in Germany concluded that the average level of human exposure to DEHP was approximately 0.0024 mg/kg B.W/day, much below the current “No Observed Adverse Effect Level” (NOAEL) adopted by the European Food Safety Authority for DEHP at 5 mg/kg/day [39]. However, this is not adequate grounds for dismissing further study and regulation. DEHP levels, amongst other phthalates, are likely to be underestimated through monoester urine screening and the effects of various phthalates are thought to be cumulative [8, 40, 41]. Moreover, studies in human populations are increasingly associating phthalate exposure with adverse effects, highlighting the importance of a more complete and widespread understanding of the behavior, potential for bioaccumulation, and the adverse effects of phthalates in human populations. 

The most widely studied adverse effect of phthalate exposure thus far suggests a potential disturbance in the development and function of reproductive organs through endocrine disruption [42–55]. Exposure of animals to high levels of phthalates results in a well-described change: the testis decrease in weight with atrophy of seminiferous tubules, progressive degeneration of germ cells, Sertoli cell dysfunction, and hormonal disruption in Leydig cells [19–21, 56–61]. Prepubertal and pubertal males appear to be more vulnerable at lower doses and a shorter duration of exposure leading to the changes described [62]. 

In developing male rats, phthalate metabolites were found to inhibit fetal testicular testosterone biosynthesis through changes in gene expression of enzymes and proteins necessary for fetal Leydig cell function [42–45]. This is especially prevalent with exposure to DEHP, dibutyl phthalate (DBP), and butyl benzyl phthalate (BBP) and results in anatomical anomalies consistent with the disruption of androgen-dependent development [63, 64]. Observed changes include cryptorchidism, hypospadias, reduced sperm production, permanent retention of nipples, atrophy or agenesis of sex accessory organs, and decreased anogenital distance [64, 65]. The severity and frequency of these manifestations appear to be dose-dependent, with the most distorted malformations occurring at 750 mg DEHP/kg/day, and subtler manifestations at as low as 6 mg/kg/day in animal models [64, 66]. A recent study in male infants was the first of its kind in expressly demonstrating such an association in humans. Swan et al. found a significant correlation between increased levels of phthalates in maternal urine and a decreased (feminized) anogenital distance in their male offspring, suggesting that prenatal exposure to phthalates may be of real consequence for people [46]. 

Adult female rats have traditionally appeared less sensitive to phthalate exposure; however, there is evidence that at high levels (2000 mg/day), they develop reduced serum estradiol levels, prolonged estrous cycles, and at times may cease to ovulate [51]. Phthalate exposure has also been associated with a delay in the onset of puberty, a decrease in fertility, and an increased incidence of mid-gestation spontaneous abortion [50, 65, 67]. Metabolites are believed to target the ovary, where suppression of aromatase enzyme activity limits the synthesis of estradiol. Additionally, there is evidence to suggest phthalate exposure may have a teratogenic effect, resulting in both visceral and skeletal anomalies in animal models [4, 52, 68]. 

Given the emerging literature in this field, a series of observational studies have been undertaken in human populations. Recognizing that trials are not possible with humans—as exposing individuals or populations to potentially toxic compounds is unethical—more prolonged and academically challenging observational studies of cohorts found to be exposed is the primary method used to draw conclusions about associations between human exposures and health outcomes. Though unable to delineate causal relationships, preliminary research has associated phthalate exposure with reduced semen quality, endometriosis, shorter pregnancy duration, and reduced anogenital distance in males [26, 53, 54, 69–71]. More recent evidence, however, has demonstrated a definitive link between “DEHP concentration in ambient air and the adverse effects in sperm motility and chromatin DNA integrity [72].” Given the widespread use of compounds containing phthalates, the implications for reproductive toxicity are concerning. 

Beyond reproductive outcomes, there has been much interest in the link between phthalate exposure and allergy and asthma symptoms in children, as well as the proposed association with an increased waist circumference and BMI [73–79]. Despite these emerging concerns, manufacturers are not obligated to include phthalates on the list of ingredients for children's products sold in Canada. Finally, it is not known whether the toxic effect of phthalates is dose dependent and whether there is a consistent threshold level where toxicity is manifest. 

In this study, approved by the Health Research Ethics Board at the University of Alberta, we endeavored to increase the understanding of the behavior of phthalate compounds by assessing human excretion of various common members of the phthalate family into each of three body fluids: blood, urine, and sweat. Both parent compounds and their metabolites were studied. 

## 3. Methods

### 3.1. Participant Recruitment

9 males and 11 females with mean ages 44.5 ± 14.4 years and 45.6 ± 10.3 years, respectively, were recruited to participate in the study after appropriate ethical approval was received from the Health Research Ethics Board of the University of Alberta. 10 participants were patients with various clinical conditions and 10 were otherwise healthy adults. Participants with health issues were recruited from the first author's clinical practice by invitation and both healthy and sick individuals were selected as samples of convenience by availability, wish to participate, and ease of contact. Each participant in the study provided informed consent and volunteered to give one 200 mL random sample of blood, one sample of first morning urine and one 100 mL sample of sweat. Demographic and clinical characteristics of all research participants are provided in Table 1.

### 3.2. Samples Collection

All blood samples were collected at one DynaLIFE laboratory site in Edmonton, AB, Canada with vacutainer blood collection equipment (BD Vacutainer, Franklin Lakes, NJ 07417, USA) using 21-gauge stainless steel needles which were screwed into the “BD Vacutainer One-Use Holder” (REF 364815). The 10 mL glass vacutainer was directly inserted into the holder and into the back end of the needle. This process and the use of glass blood collection tubes were used to prevent contamination. Blood was collected directly into plain 10 mL glass vacutainer tubes, allowed to clot, and after 30 minutes was centrifuged for 10 minutes at 2,000 revolutions per minute (RPM). After serum was separated off, samples were picked up by ALS Laboratories (about 3 kilometres from the blood collection site) for storage pending analysis. When received at ALS, serum samples were transferred to 4 mL glass vials and stored in a freezer at −20°C, pending transfer to the analytical laboratory. We chose to analyze phthalates in serum rather than in whole blood, based on the fact that the matrix effect of serum is much lower than whole blood. 

For urine collection, participants were instructed to collect a first morning midstream urine sample directly into a provided 500 mL glass jar container with Teflon-lined lid on the same day that blood samples were collected. Urine samples were delivered by the participants directly to ALS Laboratories, Edmonton. Samples were transferred to 4 mL glass vials and stored in a freezer at −20°C, pending transfer. 

For sweat collection, participants were instructed to collect perspiration from any site on their body directly into the provided 500 mL glass jar container with Teflon-lined lid—by placing the jar against their prewashed skin (with toxicant-free soap, water, and nonplastic brush) when actively sweating or by using a stainless steel spatula against their skin to transfer perspiration directly into the glass jar (stainless steel—made up primarily of iron, chromium, and nickel—was chosen as it is the same material as the needles used in standard blood collections and is reported not to off-gas or leach at room or body temperature). In excess of 100 mL of sweat was provided in all but one case. Each of the glass bottles used for sampling in this study was provided by ALS laboratories and had undergone extensive cleaning and rinsing. The containers were deemed appropriate for sweat collection with negligible risk of contamination: laboratory-grade phosphate-free detergent wash; acid rinse; multiple hot and cold deionized water rinses; oven dried; capped and packed in quality-controlled conditions. Sweat was collected within 1 week before or after collecting the blood and urine samples. No specifications were given as to how long sweating had commenced before collection. 10 participants collected sweat inside a dry infrared sauna, 7 collected inside a steam sauna, and 3 collected during and immediately after exercise—no specific instruction was given regarding the type or location of exercise. Participants were educated about the research and phthalate sources and were asked to meticulously avoid exposure to any potential sources of phthalates (and other toxicants) around the time of collection. Sweat was delivered by the participants directly to ALS laboratories. Samples were transferred to 4 mL glass vials and stored in a freezer at −20°C, pending analysis. No preservatives were used in the jars provided for sweat and urine collection, nor in the serum storage vials. 

### 3.3. Laboratory Method Description

The list of compounds tested for in this phthalate study—both parent and metabolites—are listed in Table 2. As parent compounds are metabolized prior to urine excretion, they were tested for in both sweat and blood but not in urine; metabolites were sought in all three fluid compartments—blood, urine, as well as sweat. High performance liquid chromatography/mass spectrometry (HPCL/MS) was used to determine phthalate metabolite concentrations while gas chromatography/mass spectrometry (GC/MS) was used to assess parent phthalates. 

The methodology for determining parent phthalates in serum and sweat was as follows. Samples (serum and sweat) were weighed into glass tubes (ca 1 g) and 1 mL of acetonitrile was added in order to precipitate serum and plasma proteins. The resulting mixture was serially extracted twice with 5 mL portions of hexane  :  dichloromethane (8 : 1, v/v) using sonication as per Colon et al., [55]. The resulting extracts were combined and concentrated to 200 microliters. Analysis was performed using gas chromatography/selected ion-monitoring mass spectrometry. Ions monitored include: dimethylphthalate (DMP), *m*/*z* 194/163; diethylphthalate (DEP), *m*/*z* 177/222; dibutylphthalate (DBP), *m*/*z* 223/278/205; benzylbutylphthalate (BBP), *m*/*z* 206/238; dicyclohexylphthalate (DCHP), *m*/*z* 249/330; diethylhexylphthalate (DEHP), *m*/*z* 279/390; disonylphthalate (DiNP), *m*/*z* 293/418. Prior to analysis all extracts were diluted 1 : 4 with hexane. Quantitation was performed using external standard calibration. Quality control was measured by analyzing method blanks, analyzing water fortified with the analytes of interest, as well as calf serum samples. The recovery of the phthalates from fortified water was 87–108% with a relative standard deviation of 1.9 to 9.0%. The relative percent difference for calf serum was 0.7 to 12% with the exception of DMP which was 23%. Instrument detection limits were determined to be 8 ng/g. 

Serum, sweat, and urine were analyzed for phthalate metabolites following the general procedures established by the US Centers for Disease Control and Prevention [80, 81]. Briefly, 1.0 g of serum, sweat, or urine was fortified with 10 nanograms of isotopically-labelled phthalate metabolites, 20 micrograms of 4-methylumbelliferone glucuronide, 20-micrograms of labeled 4-methylumbelliferone, 500 microliters of ammonium acetate buffer (pH 6.5), and 10 microliters of *β*-glucuronidase (*Escherichia coli* K12, Roche Biomedical). The samples were mixed and incubated at 37°C for 90 minutes to allow for the deglucuronidation of the phthalate metabolites. 

Following enzymatic hydrolysis, an aliquot (20 uL) was removed and analyzed for 4-methylumbilliferone to determine enzymatic hydrolysis efficiency. The remainder was removed and loaded onto a Zymark Rapid Trace Station for automated solid phase extraction (SPE). The 60 milligram/3 mL Oasis-HLB cartridges was conditioned with HPLC-grade methanol (2 mL) and 0.1 M formic acid (1 mL). The samples were diluted with 5 mL of 0.1 M formic acid and loaded onto the SPE cartridge at a rate of 0.5 mL/min. The cartridge was washed with water (1 mL) and 10% methanol in water (2 mL) at a flow rate of 1 mL/min. The phthalate metabolites and bisphenol A were eluted with 1.0 mL of acetonitrile at a flow rate of 0.5 mL/min. The eluate was evaporated to dryness under a stream of dry nitrogen and the residue reconstituted in 85% methanol in water (200 microliters) and transferred to glass autosampler vials prior to analysis. Prior to analysis, labeled sodium perfluoro-1-octanesulfinate (5 nanograms) was added as an internal standard. 

Quality control for phthalate metabolites was maintained by analyzing a method blank (calf serum) and two spiked calf serum samples along with every 17 samples. The calf serum samples were spiked with phthalate metabolites at 20 ng/mL. The detection limit (0.2 ng/mL) for phthalate metabolites was based upon our lower calibration standard (0.5 ng/mL) which gave an instrument signal to noise response of 3 : 1. Analyses were performed using isotope dilution liquid chromatography/mass spectrometry. An API 4000 liquid chromatograph/tandem mass spectrometer was employed for the analysis of phthalate metabolites. 

## 4. Results & Discussion 

Of the 7 parent compounds tested in 19 sera and 18 sweat samples, only DBP and DEHP were detected at all. DBP was detected in 16/19 sera and 4/18 sweat samples. In 3/4 of the participants where DBP was detected in sweat, this parent phthalate was undetectable in their sera. DEHP was detected in 2 sera and 11 sweat samples, yet out of the 11 individuals who were positive for DEHP in sweat, none had DEHP detected in their serum samples. The percentage detection of the parent compounds in human serum and sweat and their frequency distributions are given in Tables 3 and 4, respectively. No attempt was made to quantitate the parent compounds in the urine samples. The distinctive findings whereby the parent phthalates are detected in sweat but not in sera may be due to the fact that these compounds have sequestered in peripheral tissues and are mobilized during perspiration, but this explanation remains speculative. 

The phthalate metabolites MEP, MiBP, and MEHP were detected in all samples of serum (*n* = 19), urine (*n* = 20), and sweat (*n* = 18), (the *n*-values differ for the differing body fluids as there were insufficient amounts of serum/sweat for testing in three samples). The percentage detection of the phthalate metabolites in the three body fluids and the frequency distributions of MEP, MiBP, and MEHP in the 3 body fluids are given in Tables 5 and 6, respectively. No phthalate metabolites other than MEP, MiBP, and MEHP were detected in the serum and sweat samples. For the 17 participants who had matched serum, urine, and sweat data for MEP, MiBP, and MEHP, we calculated the ratio of their concentrations in sweat to urine (S/U ratio) and found the following median values: MEP: 0.3, MiBP: 1.4, and MEHP: 4.6. This is suggestive of MEHP being more efficiently excreted in sweat, followed by MiBP, and urine being the best pathway of elimination of MEP. The sweat contribution to phthalate excretion may indicate release of bioaccumulated phthalates from storage sites or may originate from circulating phthalates. 

Interestingly, 5 other phthalate metabolites were detected in the urine samples, with MBzP, MEHHP, and MEOHP found in all 20 samples, MMP in 8 samples, and MCHP in 7 samples. Various phthalate metabolites including MiBP, MCHP, and others were found exclusively in urine with none evident in sera or sweat. It is hard to conclude much from this other than these compounds are commonly found in people, and that they are excreted. General population figures for selected phthalate metabolites from the National Health and Nutrition Examination Survey (NHANES) data are provided in Table 7. Mean levels for urinary MEHP and MEP are considerably higher in our study than is found in the NHANES data. This would suggest that the exposure was higher in our sample, or possibly that some participants in our sample—perhaps those with illness—differed in their ability to metabolize or excrete phthalates. The fact that a high level of consistency of phthalate results exists between individuals suggests that contamination of skin at the time of collection is not likely to have been the source of detected phthalates.

As all participants had evidence of potentially toxic metabolite MEHP, the parent compound DEHP appears to be a ubiquitous contaminant. DEHP and its most notable metabolite MEHP have been associated with liver toxicity, testicular atrophy, hormone disruption, and cardiotoxicity in animals; these concerns have led to the banning of DEHP in toys in some parts of the world [24]. It is thus important that both DEHP and MEHP appear to be eliminated in sweat according to our results. While MEHP is thought to be responsible for much of DEHP's toxicity, however, many of the known secondary metabolites have not yet been studied for their toxicity [24]. 

It has previously been thought that after DEHP enters the body, it is readily metabolized into various metabolites that are readily excreted, including MEHP. Accordingly, it has been surmised that without bioaccumulation, DEHP toxicity is generally associated with repeated or chronic exposure. It is noteworthy in this study, however, that (i) DEHP was found in the sweat samples of a number of participants with no evidence of this compound in their serum, and that (ii) MEHP concentrations in sweat far exceed the concentration in urine. This may suggest that there is some accrual of DEHP in the tissues which is mobilized and eliminated in perspiration. It is unknown if the MEHP sweat concentration represents discharge of this circulating phthalate metabolite or the release of bioaccumulated MEHP from storage sites, such as adipose tissue. 

## 5. Conclusion

This is the first study, to our knowledge, that examines the release of phthalates into sweat. Some parent phthalate compounds and metabolites appear to be readily excreted in sweat; others do not. As all participants had evidence of the potentially toxic metabolite MEHP, the parent compound DEHP appears to be a ubiquitous contaminant in some population groups. Considering that in a number of individuals, some phthalate compounds appeared in sweat but not in serum suggests that bioaccumulation of selected phthalate compounds such as DEHP and DBP may be occurring with uncertain human toxicity. Furthermore, the toxic metabolite MEHP appears to be well eliminated in sweat. For these reasons, there may be advantage to inducing perspiration through methods such as sauna use as a means (i) to eliminate some potentially toxic phthalates and (ii) to collect samples to possibly diagnose the presence of bioaccumulated phthalate compounds such as DEHP.

With the recognition that various persistent pollutants may be determinants of chronic illness, increasing attention is being directed toward research and study of potential techniques and interventions designed to facilitate removal of persistent toxicants from the human body [82–86]. Emerging evidence in the scientific literature suggests that various persistent pollutants may be excreted through induced thermal depuration techniques such as sauna therapy, use of steam rooms, or exercise within heated quarters [87–91]. As caloric restriction appears to mobilize toxicants from storage sites [84, 92] and the skin may act as an alternative storage compartment in the face of decreasing fat stores [92], measures to facilitate loss of adipose tissue may act synergistically to enhance toxicant mobilization through the skin. Recognizing the potentially toxic effect of DEHP and MEHP, regular depuration through sweating may offer health benefits by precluding sequelae associated with bioaccumulated phthalates and toxic metabolites.

## 6. Key Findings

- DEHP and/or its metabolite MEHP were found in all participants, suggesting that exposure to potentially toxic phthalate compounds is very common.
- Some parent phthalate compounds and some metabolites appeared to be readily excreted in sweat; others did not.
- In several individuals, DEHP was found in sweat but not in serum, suggesting the possibility of some degree of phthalate retention and bioaccumulation.
- Some toxic phthalate metabolites such as MEHP were eliminated comparatively well in sweat.
- Several phthalate metabolites were evident in urine with no evidence of the parent compound in either serum or sweat.

## Figures and Tables

**Table 1:** Participant demographics and general clinical characteristics.

Participant	Gender	Age	Clinical diagnosis	Technique used for sweat collection
1	M	61	Diabetes, obesity, hypertension	Exercise
2	F	40	Rheumatoid arthritis	Steam Sauna
3	M	38	Addiction disorder	Steam Sauna
4	F	25	Bipolar disorder	Steam Sauna
5	F	47	Lymphoma	Steam Sauna
6	F	43	Fibromyalgia	Steam Sauna
7	F	48	Depression	Steam Sauna
8	F	40	Chronic fatigue	Infrared Sauna
9	F	68	Diabetes, fatigue, obesity	Steam Sauna
10	M	49	Chronic pain, cognitive decline	Exercise
11	M	53	Healthy	Exercise
12	M	23	Healthy	Infrared Sauna
13	M	21	Healthy	Infrared Sauna
14	F	47	Healthy	Infrared Sauna
15	M	53	Healthy	Infrared Sauna
16	F	43	Healthy	Infrared Sauna
17	F	51	Healthy	Infrared Sauna
18	M	46	Healthy	Infrared Sauna
19	M	57	Healthy	Infrared Sauna
20	F	50	Healthy	Infrared Sauna

**Table 2:** Phthalate compounds tested.

Parent compounds	Corresponding metabolites
DMP (dimethyl phthalates)	MMP (monomethyl phtalate)
DEP (diethyl phthalates)	MEP (monoethyl phthalate)
DBP (dibutyl phthalates)	MBzP (mono-benzyl phthalate) MiBP (mono-iso-butyl phthalate)
BBP (benzyl butyl phthalates)	MBzP (mono-benzyl phthalate)
DCHP (dicyclohexyl phthalates)	MCHP (mono-cyclohexyl phthalate)
DEHP (di (2-ethylhexl)phthalates)	MEHP (mono(2-ethylhexyl) phthalate) MEHHP (mono-(2-ethyl-5-hydroxyhexyl)phthalate) MEOHP (mono-(2-ethyl-5-oxohexyl) phthalate)
DiNP (di-isononyl phthalates)	MINP (monoisononyl phthalate)
DOP (di-octyl Phthalate)	MOP (mono-n-octyl phthalate)

**Table 3:** Percentage of individuals with detection of parent phthalates in body compartments.

Parent compound	Serum (*n* = 19)	Sweat (*n* = 18)
DMP (dimethyl phthalate)	0	0
DEP (diethyl phthalate)	0	0
DBP (dibutyl phthalate)	84	22*
BBP (benzyl butyl phthalate)	0	0
DCHP (dicyclohexyl phthalate)	0	0
DEHP (di 2-ethylhexyl phthalate)	10	61**
DiNP (di-isononyl phthalate)	0	0
DOP (di-octyl Phthalate)	0	0

*In 3/4 of these participants where DBP was detected in sweat, this parent phthalate was not detectable in their serum samples.

**In all 11 individuals who are positive for DEHP in sweat, none of these had DEHP detected in their serum samples.

**Table 4:** Distribution of parent phthalate concentrations in serum (SE) and sweat (SW) (*μ*g/g).

	SE-DBP	SW-DBP	SE-DEHP	SW-DEHP
*n*	19	18	18	18
Mean	35.1	*	*	49.9
Std. Dev.	28.3	*	*	133
Median	37.6	*	*	15.5
Range	<8–79.0	<8–58.6	<8–35.0	<8–576

*For the 18 individuals who had their sweat tested for DBP, only 4 were above detection limit (8 *μ*g/L). Thus mean, SD and median are not reported for SW-DBP. Similarly for the 18 serum samples tested for DEHP only 2 were above the detection limit and mean, SD, and median are not reported.

**Table 5:** Percentage of individuals with detection of phthalate metabolites in body compartments.

Metabolites	Serum	Urine	Sweat
MMP (monomethyl phtalate)	0	40	0
MEP (monoethyl phthalate)	100	100	100
MiBP (mono-iso-butyl phthalate)	100	100	100
MBzP (mono-benzyl phthalate)	0	100	0
MCHP (mono-cyclohexyl phthalate)	0	35	0
MEHP (mono(2-ethylhexyl) phthalate)	100	100	100
MEHHP (mono-(2-ethyl-5-hydroxyhexyl)phthalate)	0	100	0
MEOHP (mono-(2-ethyl-5-oxohexyl) phthalate)	0	100	0
MOP (mono-n-octyl phthalate)	0	0	0
MINP (monoisononyl phthalate)	0	0	0

**Table 6:** Distribution of phthalate metabolite concentrations in serum (SE), sweat (SW), and urine (UR) (*μ*g/g).

	SE-MEP	SW-MEP	UR-MEP	SE-MiBP	SW-MiBP	UR-MiBP	SE-MEHP	SW-MEHP	UR-MEHP
*n*	19	18	20	19	18	20	19	18	20
Mean	5.69	91.1	535	26.1	111	122	28.2	27.3	12.4
SD	8.61	172	1560	23.9	75.3	96.6	9.65	21.4	23.7
Median	3.88	29.9	107	17.8	100	74.4	27.6	12.4	35.1
Range	0.84 –39.2	3.94 –750	6.76–6978	4.0–77	46.0–378	20.7–342	17–52.6	2.68–68.6	1.11–108

**Table 7:** Urinary phthalate metabolite levels in the general population (*μ*g/g) (national health and nutrition examination survey (NHANES) data) [3, 9].

	Above detection limit (%)	Geometric mean	95th percentile
MBzP	3	14.0	77.4
MEHP	22	3.1	18.5
MEOHP	N/A	13.6	118
MEHHP	N/A	20.4	182
MEP	0	63	1950
